# Screening Strategies to Control Antibiotic-Resistant Bacteria: An Agent-Based Model

**DOI:** 10.3390/antibiotics15090854

**Published:** 2026-09-01

**Authors:** Liat Wulffhart, Elizabeth Temkin, Yehuda Carmeli

**Affiliations:** 1 National Institute for Antibiotic Resistance and Infection Control, Israel Ministry of Health, Tel Aviv 6423906, Israel; liat.ruben@gmail.com (L.W.); lizt@tlvmc.gov.il (E.T.); 2 Gray Faculty of Medical and Health Sciences, Tel Aviv University, Tel Aviv 6997801, Israel

**Keywords:** antibiotic resistance, agent-based model, infection control

## Abstract

**Background/Objectives**: Screening for carriage of antibiotic-resistant bacteria (ARB) upon hospital admission and isolating carriers can prevent nosocomial spread of ARB. Screening typically targets patients at high risk of ARB carriage because of recent hospitalization or travel. For ARB with community spread among non-high-risk patients, screening only high-risk patients may be insufficient to control nosocomial transmission. We used an agent-based model to examine the impact of screening patients without recognized risk factors for ARB carriage on the acquisition and prevalence of ARB carriage in hospitals. **Methods**: The agents in our model of an 800-bed hospital were patients and healthcare workers (HCW). Contacts between HCW and patients or between patients may result in ARB transmission. We modelled scenarios of 1%, 5% or 20% ARB carriage prevalence upon admission among high-risk patients and ARB carriage prevalence ratios between high-risk and non-high-risk patients of 1.11, 2, 5 and 10. At baseline, screening test sensitivity was 85%, compliance with screening of high-risk patients was 80%, and isolation effectiveness was 70%. **Results**: Expanding screening to non-high-risk patients decreased the incidence of nosocomial ARB acquisitions. The impact of this screening on nosocomial acquisitions increased as greater community spread occurred. Carriage prevalence in the hospital was mainly influenced by imported cases; nosocomial transmission accounted for a minority of prevalent cases. Improving isolation effectiveness to 95% had a greater impact on ARB acquisitions than expanded screening when carriage prevalence among non-high-risk patients was low. **Conclusions**: The impact of expanded screening was small when carriage prevalence among high-risk patients was low or when the carriage prevalence ratio was high. In the scenarios modelled here, improving isolation effectiveness prevented more acquisitions than expanded screening.

## 1. Introduction

Antibiotic-resistant bacteria (ARB) are a major threat to health. Worldwide in 2021, an estimated 1.14 million deaths were attributable to ARB infections (i.e., the deaths would not have occurred if the infections had been antibiotic-susceptible) and an estimated 4.71 million deaths were associated with ARB infections (i.e., the deaths would not have occurred if there had been no infection) [1]. Typically, spread of ARB begins in healthcare settings, where selective pressure from intensive antibiotic use favors their spread. Hospitals’ efforts to limit ARB spread focus on early identification of carriers within high-risk populations and use strategies such as search-and-destroy or search-and-isolate [2,3,4].

Within two decades of spread in the healthcare setting, some ARB that began as nosocomial pathogens become widely transmitted in the community [5,6]. The most notable examples of this chain of events are penicillin-resistant *Staphylococcus aureus* [7], methicillin-resistant *S. aureus* (MRSA) [8] and extended-spectrum beta-lactamase (ESBL)-producing Enterobacterales [9]. Community transmission drastically expands the population of carriers, increasing the incidence of ARB infections [5]. Moreover, the expansion of ARB into the community and among populations without known risk factors for ARB carriage (“non-high-risk patients”) may thwart hospitals’ infection control efforts, which rely on identification of carriers, as ARB carriage among these patients escapes detection if only high-risk-populations are screened.

One option for controlling nosocomial spread of community-acquired ARB is universal screening upon admission. The advantage is enhanced detection and subsequent isolation of carriers. The disadvantages are the cost of screening and the logistical challenge of potentially needing isolation for many patients, especially difficult in settings where single-patient rooms are scarce. The effectiveness and cost-effectiveness of universal screening have been studied most extensively for MRSA [10,11,12,13,14].

During the last two decades, carbapenem-resistant Enterobacterales (CRE) have emerged as nosocomial pathogens, causing outbreaks and, in some healthcare institutions, reaching endemicity [15]. CRE are classified as an urgent threat by the US Centers for Disease Control [16] and as critical priority pathogens for drug development by the World Health Organization [17]. The European Centre for Disease Control recently issued a risk assessment that indicated a high probability of further spread of CRE in the European Union/European Economic Area, both in healthcare settings and in the community [18].

Israel has a comprehensive, nation-wide intervention in place since 2007 to control the spread of CRE [19]. While this intervention has been highly successful in preventing transmission of most CRE, newly detected cases of the CRE OXA-48-like-producing *Escherichia coli* (OXA-EC) per million population have increased 9-fold between 2017 and 2023 [20]. In those years, nationwide, the annual number of reported hospital-acquired OXA-EC cases increased from 84 to 652 and the annual number of reported cases present on admission increased from 74 to 872. In 2022–2023, 24.5% of OXA-EC cases were detected upon hospital admission in patients not included in high-risk populations for which CPE screening is indicated, i.e., they had no recent history of healthcare exposure or foreign travel, indicating community acquisition [20]. In 2024, this group comprised 34.6% of incident OXA-EC cases (Ministry of Health, unpublished data). Community transmission of OXA-EC has also been reported in Europe [21]. Expansion of screening upon hospital admission to identify non-high-risk carriers might slow the nosocomial spread of OXA-EC.

Here, we used an agent-based model to explore the effect of expanding screening for ARB carriage among non-high-risk patients upon hospital admission. As we were interested in the questions of spread of ARB and the burden of isolation, we chose as outcomes the incidence of nosocomial ARB acquisition and the prevalence of ARB carriers in the hospital.

## 2. Results

In our agent-based model of an 800-bed hospital, we examined the effect of three parameters on the incidence of nosocomial ARB acquisitions and the prevalence of ARB carriers in the hospital: (1) the prevalence of ARB carriage upon admission among non-high-risk patients, (2) the prevalence of ARB carriage upon admission among high-risk patients, and (3) the percentage of non-high-risk patients who are screened for ARB carriage upon admission. For the incidence outcome only, we examined the impact of two additional parameters: effectiveness of carrier detection and isolation.

### 2.1. Impact of Screening on the Incidence of ARB Acquisitions, Depending on ARB Carriage Prevalence Upon Admission Among Non-High-Risk Patients

**Figure 1:**
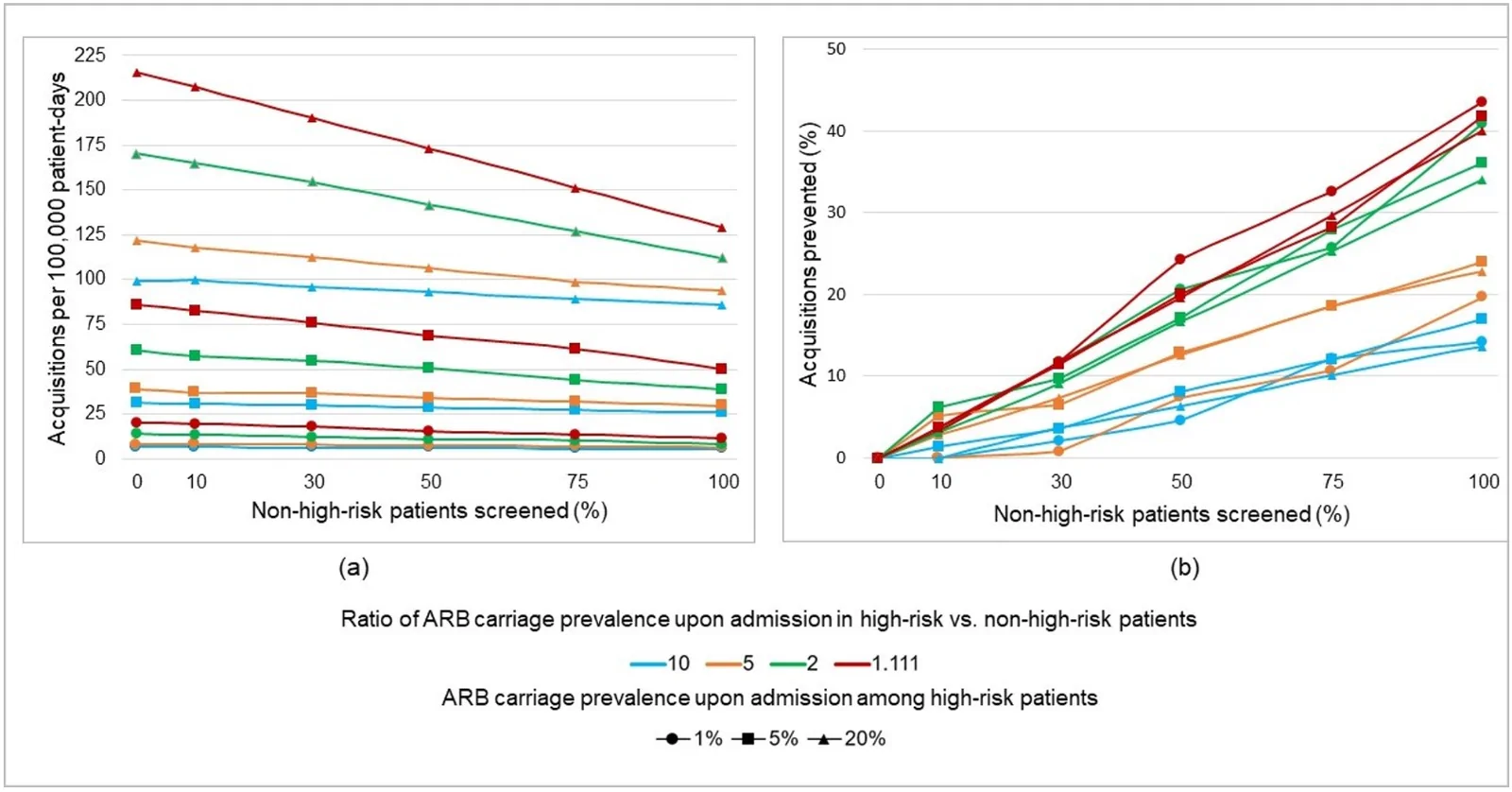
(**a**) Incidence of antibiotic-resistant bacteria (ARB) acquisition and (**b**) percentage of acquisitions prevented by expanding screening of non-high-risk patients (compared to no screening of non-high-risk patients) under three scenarios of ARB carriage prevalence upon admission among high-risk patients: 1%, 5% and 20%. Lines represent the ratio of ARB carriage prevalence between high-risk patients and non-high-risk patients. Test sensitivity is 85%, compliance with screening of high-risk patients is 80% and effectiveness of isolation of identified carriers is 70%.

Figure 1a and Table S1 show the incidence of ARB acquisition under three scenarios of ARB prevalence among high-risk patients (1%, 5% and 20%), and at varying carriage prevalence ratios between high-risk and non-high-risk patients. We modeled a scenario where the screening test has 85% sensitivity and the compliance with screening of high-risk patients is 80%.

At a given ARB carriage prevalence upon admission among high-risk patients, as prevalence among non-high-risk patients increases (i.e., the carriage prevalence ratio falls), the greater the impact of expanding screening. For example, when carriage prevalence among high-risk patients is 1% and carriage prevalence among high-risk and non-high-risk patients is almost equal (1% vs. 0.9%, [a prevalence ratio of 1.11], Figure 1a, red line with circle markers), nine acquisitions per 100,000 patient-days are prevented by universal screening (100% of non-high-risk patients are screened) compared to no screening of non-high-risk patients. This decrease corresponds to preventing 44% of acquisitions (Figure 1b, red line with circle markers, and Table S2). In contrast, when carriage prevalence upon admission among high-risk patients is 1% and carriage prevalence among non-high-risk patients is low (1% vs. 0.1%, [a prevalence ratio of 10], Figure 1a, blue line with circle markers), only 1 acquisition per 100,000 patient-days is prevented by universal screening. This decrease corresponds to preventing 14% of acquisitions (Figure 1b, blue line with circle markers).

### 2.2. Impact of Screening on the Incidence of ARB Acquisitions, Depending on ARB Carriage Prevalence Upon Admission Among High-Risk Patients

At a given carriage prevalence ratio, as prevalence among high-risk patients increases, screening of more non-high-risk patients prevents more acquisitions (Figure 1a), but the percent reduction is similar (Figure 1b). For example, when prevalence among high-risk patients is 1% and the carriage prevalence ratio is 5, i.e., the prevalence among non-high-risk patients is 0.2% (Figure 1a, orange line with circle markers), two acquisitions per 100,000 patient-days are prevented when universal screening of non-high-risk patients is performed. This decrease corresponds to prevention of 20% of acquisitions (Figure 1b, orange line with circle markers). In contrast, when prevalence among high-risk patients is 20% and the carriage prevalence ratio is 5, i.e., the prevalence among non-high-risk patients is 4% (Figure 1a, orange line with triangle markers), 28 acquisitions per 100,000 patient-days are prevented by universal screening. This decrease corresponds to a similar percentage of acquisitions prevented- 23% (Figure 1b, orange line with triangle markers).

### 2.3. Impact of Screening on the Daily Hospital-Wide Prevalence of ARB Carriers

**Figure 2:**
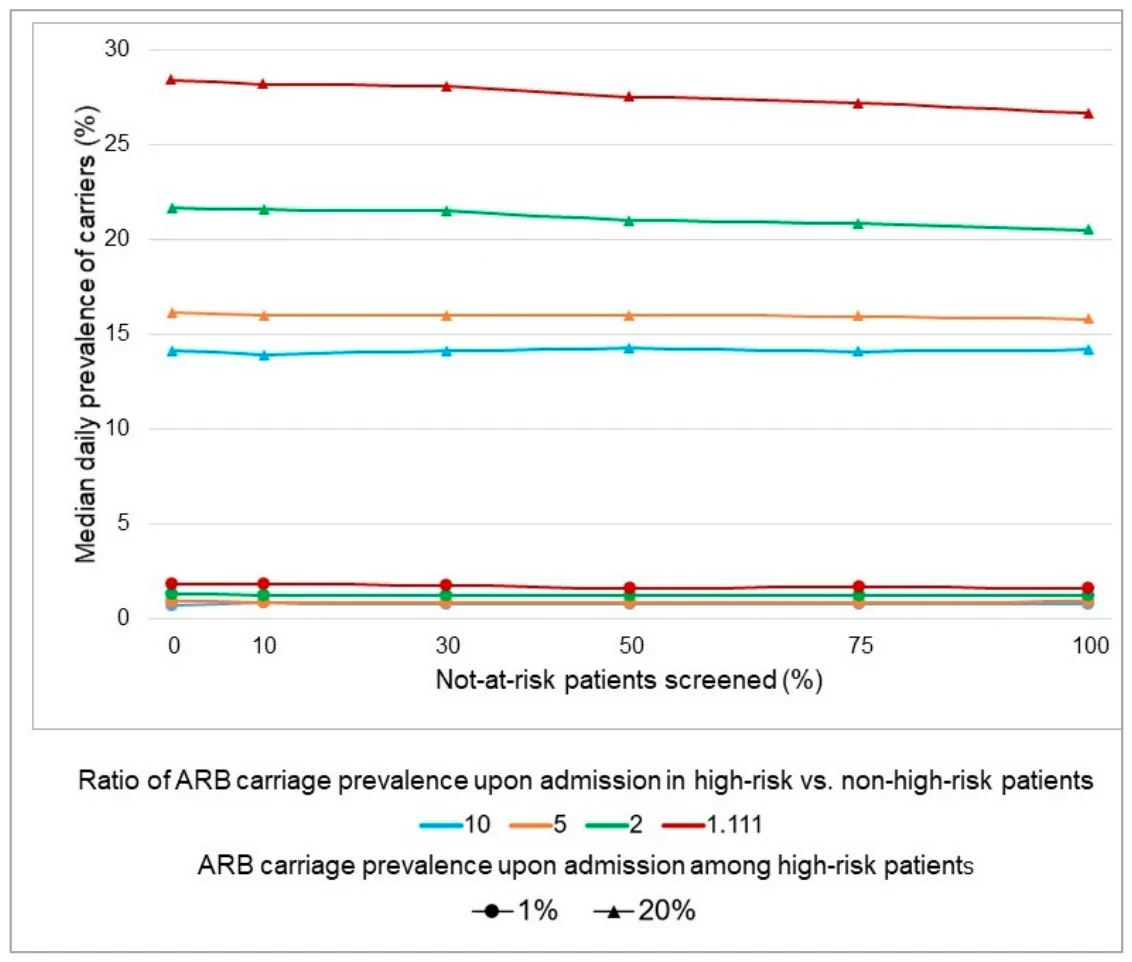
Prevalence of ARB carriers (detected and undetected) in the hospital under two scenarios of carriage prevalence among high-risk patients upon admission: 1% and 20%. Lines represent the ARB carriage prevalence ratio between high-risk patients and non-high-risk patients. Test sensitivity is 85%, compliance with screening of high-risk patients is 80% and effectiveness of isolation of identified carriers is 70%.

Figure 2 and Table S3 show the effect of the intensity of screening of non-high-risk patients on the median daily hospital-wide prevalence of ARB carriers (both detected and undetected) under two scenarios: 1% and 20% ARB carriage prevalence upon admission among high-risk patients. In both scenarios, the lines are nearly flat, indicating that greater screening of non-high-risk patients has only a minor effect on the daily prevalence in the hospital. The trend was similar regardless of carriage prevalence among non-high-risk patients upon admission. The only difference between the two scenarios is the scale. For example, in the scenario of 1% carriage prevalence among high-risk patients the daily hospital-wide prevalence of carriers at baseline (when there is no screening of non-high-risk patients) ranges from 0.7% to 1.8%. In the scenario of 20% carriage prevalence among high-risk patients, the daily hospital-wide prevalence of carriers at baseline ranges from 14.2% to 28.4%. While the lines are nearly flat, increasing the percentage of non-high-risk patients who are screened results in more carriers changing status from undetected to detected, triggering isolation requirements.

The reason that screening barely affects the daily prevalence is that the majority of prevalent cases are imported cases (i.e., patients who were already carriers upon admission). For example, in the scenario of 20% carriage prevalence among high-risk patients and a 1.11 carriage prevalence ratio (Figure 2, red line with triangle markers), with universal screening, the mean daily number of carriers is 212 (equivalent to a hospital-wide prevalence of 26.7%): 188 of them (88.7%) were imported cases (143 detected upon admission and 45 not detected) and 24 (11.3%) acquired the ARB while hospitalized. Policies regarding screening and subsequent isolation only affect the latter number. In the same scenario but with no screening of non-high-risk patients, hospital-wide prevalence rises only slightly to 28.4%: of 227 carriers, 188 of them (82.8%) were imported cases (67 detected upon admission and 121 not detected) and 39 (17.2%) acquired an ARB during hospitalization. It is important to note that without screening of non-high-risk patients, the mean daily number of hospital- acquired cases rose by 15 (62.5%).

### 2.4. Impact of Improved Screening Test Sensitivity, Compliance with Screening of High-Risk Patients and Isolation

**Figure 3:**
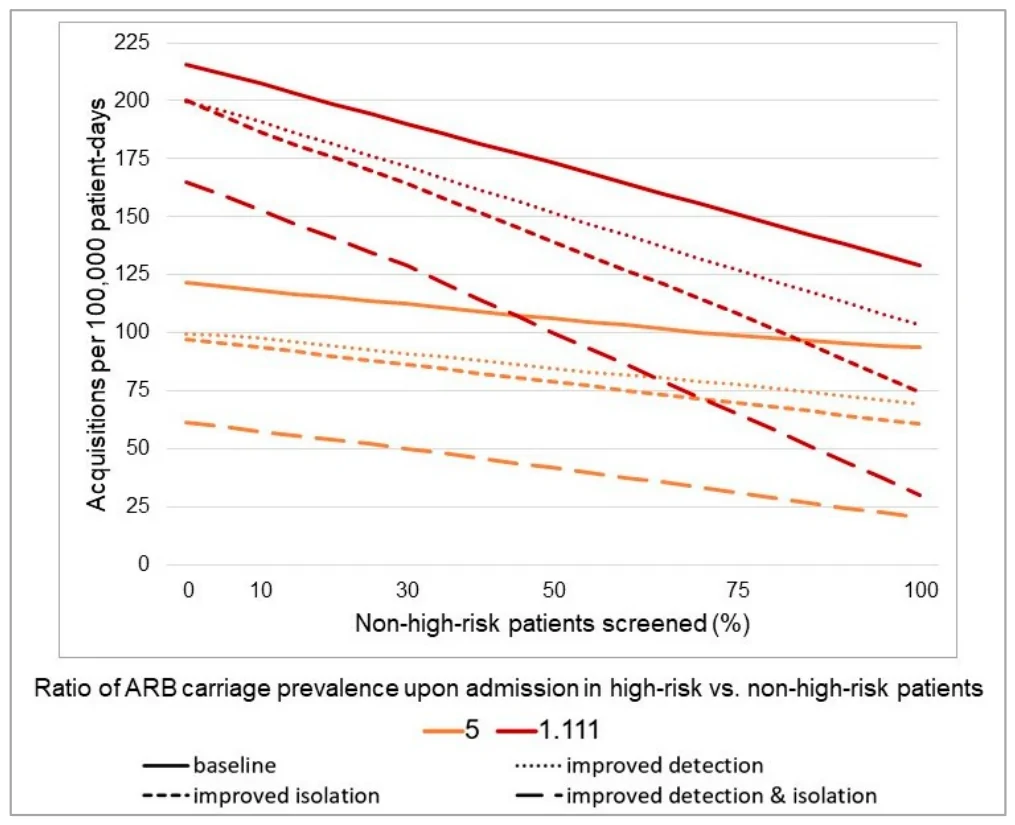
Incidence of ARB acquisitions in the scenario of 20% prevalence of antibiotic-resistant bacteria carriage among high-risk patients, with improved screening, test sensitivity, and isolation. Lines represent the ARB carriage prevalence ratio between high-risk patients and non-high-risk patients. Test sensitivity, compliance with screening of high-risk patients, and effectiveness of isolation are as follows: Baseline: 85%, 80%, 70%. Improved detection: 95%, 98%, 70%. Improved isolation: 85%, 80%, 95%. Improved detection and isolation: 95%, 98%, 95%.

We examined the effect of improving detection and isolation when ARB carriage prevalence on admission among high-risk patients is 20% and the carriage prevalence ratio is 5 or 1.11. To improve detection, test sensitivity has increased to 95% and compliance with screening of high-risk patients has increased to 98%. Effectiveness of isolation has improved to 95%. As seen in Figure 3 and Table S4, when there is no screening of non-high-risk patients, the impacts of improved detection (dotted lines) and improved isolation (small, dashed lines) are similar. As the percentage of non-high-risk patients screened increases, the impact of improved isolation surpasses that of improved detection. Improving both detection and isolation has a synergistic effect.

When carriage prevalence among high-risk patients is 20% and the carriage prevalence ratio is 5 (i.e., carriage prevalence among non-high-risk patients is 4%), improving detection, isolation, or both, with no screening of non-high-risk patients, is more effective at preventing ARB acquisitions than screening all non-high-risk patients at baseline conditions. When carriage among high-risk and non-high-risk patients is nearly equal (prevalence ratio of 1.11), improving detection and isolation are not adequate substitutes for screening of non-high-risk patients. Improving isolation and screening 60% of non-high-risk patients has a similar effect to universal screening of non-high-risk patients when isolation effectiveness is at baseline: both prevent approximately 87 acquisitions per 100,000 patient-days (a 60% reduction) compared to no screening of non-high-risk patients and baseline isolation effectiveness.

## 3. Discussion

We presented the results of an agent-based model examining the impact of an expanded screening policy (i.e., including patients without recognized risk factors for ARB carriage) on the acquisition and prevalence of ARB carriage in hospitals. We found that wider screening of non-high-risk patients decreased the incidence of nosocomial ARB acquisitions. The impact of screening increased as greater community spread occurred, i.e., carriage prevalence among non-high-risk patients approached that of patients at high risk of carriage. The effect of expanded screening on nosocomial transmission was greater as the prevalence of carriage on admission increased. We found that, given the fixed parameters of our model, the prevalence of ARB carriers in the hospital was influenced mostly by the imported cases and that nosocomial transmission accounted for a minority of prevalent cases. Our model showed that improving isolation prevented ARB acquisitions better than improving detection by enhancing both test sensitivity and compliance with screening of high-risk patients. Moreover, when carriage prevalence among non-high-risk patients was only a fifth of that in high-risk patients, improving detection or isolation with no screening of non-high-risk patients was more effective than screening all non-high- risk patients. Improving both detection and isolation had a synergistic effect.

Zahar et al. suggested that universal screening for community-acquired ARB is unnecessary as long as horizontal infection control measures, such as hand hygiene and other standard precautions, are in place to prevent transmission from undetected carriers [22]. However, when nosocomial transmission persists, additional, vertical (pathogen-directed) measures such as screening, isolation, and cohorting are required. Israeli hospitals are currently facing outbreaks of OXA-EC, with a rising incidence of nosocomial cases and an even greater increase in imported cases, despite national protocols for vertical measures [4] that successfully controlled CPE in the past [19]. This led us to question whether expanding screening to non-high-risk patients could effectively control nosocomial spread of OXA-EC.

In 2023, the prevalence of CPE carriage (including all Enterobacterales species and carbapenemases, not only OXA-EC) among hospitalized patients in Israel targeted for screening according to protocols was about 1% [23]. It is likely that CPE carriage prevalence among non-high-risk patients is currently a fraction of that among high-risk patients. Our model suggests that, at these levels, the impact of expanded screening of non-high-risk patients would be small from a clinical perspective (at most, nine CPE acquisitions prevented per 100,000 patient-days) but meaningful from an epidemiological perspective (about a 15% reduction in acquisitions). Generalizing from our simulation of the scenario of 20% carriage prevalence among high-risk patients, improving the effectiveness of isolation and/or improving carriage detection among high-risk patients would be a better strategy for preventing nosocomial transmission of OXA-EC than screening non-high-risk patients. In contrast, globally, the carriage prevalence of ESBL-producing Enterobacterales in 2016–2020 was estimated as 26% in both healthcare and community settings [24]. Hospitals with uncontrolled transmission of these ARB could benefit from widespread screening of patients with and without defined risk factors for carriage (i.e., recent contact with the healthcare system or travel) in addition to improving the effectiveness of isolation and test sensitivity.

Previous studies have examined the effect of universal screening for ARBs. In the STAR*ICU trial, all patients in 10 intervention intensive care units (ICUs) and 8 control ICUs were screened upon admission and then weekly for MRSA and vancomycin-resistant enterococcus (VRE) carriage. Patients in the intervention units who screened positive were placed on contact precautions and all other patients were cared for using gloves only. While screening detected a hidden reservoir of carriers, the incidence of MRSA and VRE acquisition did not differ between groups, which the researchers attributed to the delay in reporting of screening results (and thus implementation of contact precautions) and to imperfect compliance with both contact precautions and gloving [25]. Likewise, serial point prevalence studies in Chicago ICUs found no change in the prevalence of MRSA carriage before and after a law in Illinois mandated universal MRSA screening (and contact precautions for carriers) in ICUs [26]. Studies in Veterans Affairs hospitals [13] and in England [27] finding that universal MRSA screening reduced MRSA infections may have been confounded by the concurrent implementation of other infections control measures [26,28]. Modelling studies have shown that universal screening for MRSA is not cost effective and less preferable than screening only patients admitted to specialty wards where the risk if MRSA infection is high [10], or cost saving only when carriage prevalence upon admission is high (>5.5%) [11].

We found that screening of non-high-risk patients had only a very small impact on the mean daily prevalence of ARB carriers in the hospital. Even when nosocomial ARB transmission is occurring, patients who were carriers upon admission comprise the vast majority of prevalent cases. A study of daily *S. aureus* screening in one medical ICU supports our finding: 5.7% of patients were MRSA carriers, all of whom were positive upon admission. (In that study, no nosocomial *S. aureus* transmission was detected.) Thus, the main effect of screening non-high-risk patients is not a drop in prevalence of carriers in the hospital, but the detection of more patients who require isolation, potentially straining hospital resources. Under-screening will create the illusion that the magnitude of carriage is smaller but will carry the cost of increased nosocomial transmission.

Our simulation addressed the incidence of ARB colonization, not of infection. Preventing colonization has unique importance: it stops the exponential spread [29,30] of antibiotic resistance. Since, in almost all cases, colonization precedes infection [31], the effect of preventing colonization will eventually translate to exponential prevention of ARB infections. The proportion of carriers who will progress to infection is determined by the virulence of the organism, patient characteristics, and treatments received (e.g., invasive procedures, immunosuppression, and antibiotics). For example, Huang et al. reported that 29% of patients with MRSA detected during hospitalization developed a MRSA infection within 18 months [32]. We reported a 0.3–9.4% risk of progression from CPE carriage to CPE bloodstream infection within one year, based on setting (e.g., community vs. oncology ward) [33]. To expand the finding of our simulation to estimate the effect of screening on infection prevention, the specific pathogens and patients involved must be considered.

Our study has several limitations. First, our model has fixed parameters, such as a minimum of 80% compliance with screening of high-risk patients and a minimum of 70% effectiveness of isolation, that may not be representative of all hospitals. We performed sensitivity analyses for ARB carriage prevalence, compliance with screening, test sensitivity and isolation effectiveness, but not for other parameters such as frequency of hand hygiene, ARB transmission probability, ward design, and length of stay. Thus, the generalizability of our results is limited. Future studies should examine the impact of characteristics of the hospital and the health care system on ARB transmission. Second, in our model, increased nosocomial transmission does not affect the number of imported cases. In fact, over time, transmission affects prevalence both directly (as measured in our model) and indirectly by increasing the load of imported cases through readmission of patients who acquired ARB in the hospital. Third, all wards in our model have the same characteristics. In reality, characteristics that affect transmission, such as the number of healthcare worker contacts with patients (reflecting patient acuity), crowding, and nurse-to-patient ratio, differ between ward types. Fourth, we tested a scenario in which effectiveness of isolation was increased from 70% to 95%; the latter may be achievable, especially in settings with high carriage prevalence, only by strict cohorting of patients and complete separation of staff and equipment for carriers and non-carriers.

In conclusion, in our model of ARB with community spread, expanding screening upon hospital admission to patients without recognized risk factors for ARB carriage limited nosocomial ARB acquisitions. The impact was small when carriage prevalence among high-risk patients was low or when the carriage prevalence ratio was high; in these conditions, improving isolation effectiveness prevented more acquisitions than expanded screening, and expanded screening has only a slight effect on prevalence of carriers in the hospital. Since different healthcare settings will differ in various parameter values, our results are not generalizable to all healthcare systems without parameter adaptations. A cluster-randomized trial comparing universal screening for ARB carriage upon admission to screening only patients at high risk of ARB carriage, conducted in hospitals with different carriage prevalence, is necessary to confirm our findings; previous such trials conducted for MRSA did not favor universal screening.

## 4. Materials and Methods

### 4.1. Model Overview

We constructed an agent-based model in GAMA 1.8.1 (http://gama-platform.org/). The model was based in part on our previous work modeling the effect of antibiotics on ARB transmission [34]. The agents in the simulation consisted of patients and health care workers (HCW). Contacts between patients or between HCW and patients may result in transmission.

**Figure 4:**
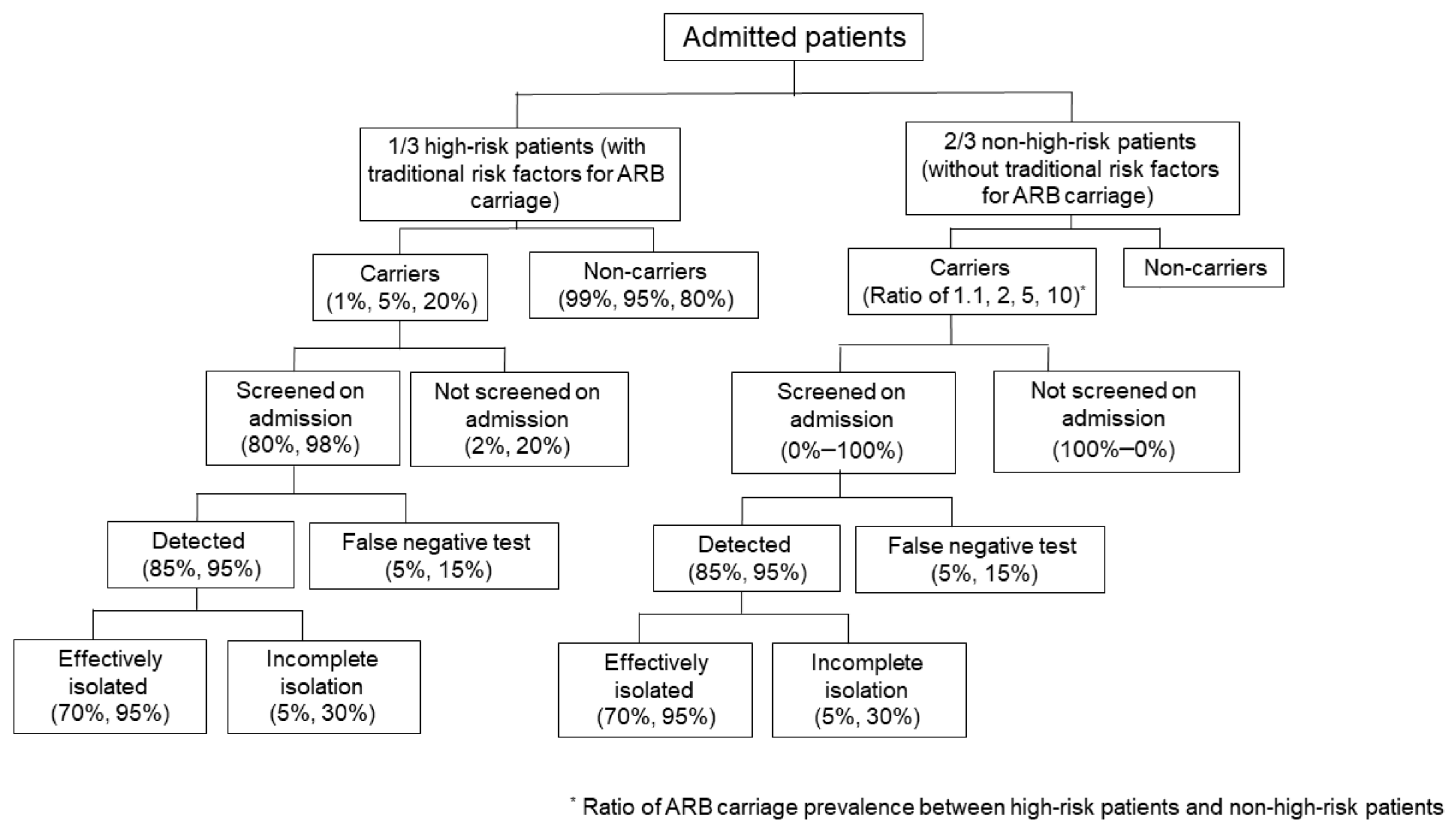
Varying parameters used in the simulation.

Fixed model parameters are shown in Table 1 and varying parameters are shown in Figure 4. Values were based on the literature or, when not available in the literature, on data from our hospital, Tel Aviv Sourasky Medical Center (Tel Aviv, Israel), in 2014–2016, or national data from Israel’s Ministry of Health.

Upon admission to the hospital, patients may belong to either the “high-risk” group or the “non-high-risk” group according to whether they have defined risk factors for ARB carriage (such as recent hospitalization or foreign travel, transfer from a long-term care facility, or transfer from a high-risk ward with the hospital). The percentage of patients in the high-risk group who are screened for ARB carriage on admission is a varying parameter with values of 80% or 98%. The percentage of non-high-risk patients who are screened can be varied between 0% and 100%. Patients are randomly assigned to a ward, bed, length of stay (LOS) and whether they receive antibiotics. LOS varies based on carriage status. ARB carriers remain colonized for the duration of their hospital stay [42,43]. Patients who screen positive for ARB carriage are isolated. The probability of transmission decreases for patients in isolation. Isolation includes physical separation of carriers from non-carriers, healthcare workers’ use of gloves and gowns, dedicated staff for carriers, dedicated equipment for carriers or disinfection or sterilization of equipment shared between carriers and non-carriers, and enhanced cleaning of the environment surrounding carriers. Compliance with and effectiveness of all aspects of isolation is a varying parameter with values of 70% or 95%. Screening after admission is carried out in 2% of patients per day. (The count of nosocomial ARB acquisitions includes those that are detected by screening after admission and those that go undetected.) For patients randomly assigned to receive antibiotics, treatment is for ten days or until discharge. Antibiotic treatment can increase both susceptibility (risk of acquisitions among non-carriers) [44,45] and infectiousness (risk of transmission among carriers) [46,47]. In our previous study, we modelled increases of 1–4 folds [34]; here, we assumed a 2-fold increase in risk of ARB acquisition and transmission among patients on antibiotics.

Ward HCWs are assigned to wards and beds. General HCWs are not assigned to a specific ward and can have contact with patients from all over the hospital. At every contact between HCW and patient, transmission of ARB can occur in either direction (from HCW to patient or vice versa) with certain probabilities. When a HCW becomes a transient ARB carrier after contact with a colonized patient, performing hand hygiene reverses the carriage state and prevents the HCW from transmitting ARB to subsequent patients. Patients can also transmit ARB directly to an adjacent patient with a certain probability per day. Probabilities of transmission and of hand hygiene are displayed in Table 1.

### 4.2. Simulations

To evaluate the effect of screening non-high-risk patients upon admission on the incidence of ARB acquisition, we examined three scenarios of ARB carriage prevalence upon admission among high-risk patients: 1%, 5%, and 20% (Figure 1). For each of these three scenarios, we simulated 24 combinations of: (1) percentage of non-high-risk patients who were screened on admission (six values: 0%, 10%, 30%, 50%, 75%, 100%), and (2) the ratio of ARB carriage prevalence between high-risk patients and non-high-risk patients. We used four different values for this ratio: 1.11, 2, 5 and 10. For example, when the prevalence in high-risk patients is 1%, the four ratios correspond to prevalence in non-high-risk patients of 0.9%, 0.5%, 0.2% and 0.1%. Screening of more than 0% but less than 100% of non-high-risk patients could represent either partial compliance with the screening protocol or a policy to screen non-high-risk patients only upon admission to certain wards. For each of the 24 combinations, we presented the average of 100 simulations. The time period for each simulation was two years, where ARB circulation in the hospital became established during the first year and we present the results from the second year.

For two scenarios of carriage prevalence upon admission among high-risk patients, 1% and 20%, we performed the same procedure to evaluate the effect of screening on the daily prevalence of ARB carriers in the hospital. In the scenario of 20% prevalence, we also examined the impact on ARB acquisition incidence when there are improvements in screening test sensitivity (e.g., by ensuring adequate sampling) compliance with screening of high-risk patients on admission, and effectiveness of isolation of carriers are improved.

## Figures and Tables

**Table 1:** Fixed parameters used in the simulation. TASMC refers to values derived from data from Tel Aviv Sourasky Medical Center. MOH refers to values derived from data from Israel’s Ministry of Health.

Parameter	Value	Remarks/References
**Hospital**		
Number of beds	800	
Number of wards	20	40 beds per wards. All wards are moderate acuity medical-surgical wards.
**HCW**		
Ward HCW	100	5 per ward; each is responsible for 8 beds
General HCW	80	
Ward HCW contacts with patients	4 per hour	[35]
General HCW contacts with patients	3 per hour	[35]
**Hand hygiene probability**	50%	[36] Hand hygiene opportunities are before and after patient contact
**Patients**		
Percentage of patients at high risk of ARB carriage upon admission	33.30%	MOH
LOS (days) for patients not screened or with a negative screen on admission, mean (SD)	6.3 (11.7)	TASMC, log-normal distribution; consistent with European data [37]
LOS (days) for patients with a positive screen on admission, mean (SD)	11.9 (18.7)	TASMC, log-normal distribution
Additional LOS for patients who acquire ARB during hospitalization.	1.5	TASMC, original LOS is multiplied by 1.5
Antibiotic treatment probability on admission	40%	TASMC
Screening percentage after admission (per day)	2%	TASMC
**Transmission probabilities**		Cited studies included patients receiving and not receiving antibiotics; we chose lower values as the baseline transmission probability for patients not on antibiotics [34]
Contamination of a HCW from a colonized patient, per contact	5%	[38]
Transmission from a contaminated HCW to a susceptible patient, per contact	2.50%	[39]
Transmission from a colonized patient to a susceptible adjacent patient, per day	0.50%	Based on studies that estimated risk of ARB acquisition among patients in rooms whose prior occupant carried ARB [40,41]
**Parameters affecting transmission risk**		
Antibiotics’ effect on risk that ARB carrier will transmit	2	Baseline transmission probability is multiplied by 2
Antibiotics’ effect on risk that ARB non-carrier will acquire	2	Baseline transmission probability is multiplied by 2

HCW: healthcare worker, ARB: antibiotic-resistant bacteria, LOS: length of stay.

## Data Availability

Values used to create the graphs can be found in the Supplementary Materials.

## Supplementary Materials

The following supporting information can be downloaded at: https://www.mdpi.com/article/10.3390/antibiotics15090854/s1, Table S1: Data for Figure 1a; Table S2: Data for Figure 1b; Table S3: Data for Figure 2; Table S4: Data for Figure 3.

## Abbreviations

The following abbreviations are used in this manuscript:

ARB	Antibiotic-resistant bacteria
HCW	Healthcare workers
MRSA	Methicillin-resistant *S. aureus*
ESBL	Extended-spectrum beta-lactamase
CRE	Carbapenem-resistant Enterobacterales
OXA-EC	OXA-48-like-producing *Escherichia coli*
ICU	Intensive care unit

## References

[1] GBD 2021 Antimicrobial Resistance Collaborators (2024). Global burden of bacterial antimicrobial resistance 1990–2021: A systematic analysis with forecasts to 2050. Lancet.

[2] Vos M.C., Ott A., Verbrugh H.A. (2005). Successful search-and-destroy policy for methicillin-resistant *Staphylococcus aureus* in The Netherlands. J. Clin. Microbiol..

[3] Carmeli Y., Akova M., Cornaglia G., Daikos G.L., Garau J., Harbarth S., Rossolini G.M., Souli M., Giamarellou H. (2010). Controlling the spread of carbapenemase-producing Gram-negatives: Therapeutic approach and infection control. Clin. Microbiol. Infect..

[4] Solter E., Adler A., Rubinovitch B., Temkin E., Schwartz D., Ben-David D., Masarwa S., Carmeli Y., Schwaber M.J. (2018). Israeli national policy for carbapenem-resistant Enterobacteriaceae screening, carrier isolation and discontinuation of isolation. Infect. Control Hosp. Epidemiol..

[5] van Duin D., Paterson D.L. (2020). Multidrug-resistant bacteria in the community: An update. Infect. Dis. Clin. N. Am..

[6] Heseltine P. (2000). Has resistance spread to the community?. Clin. Microbiol. Infect..

[7] Chambers H.F. (2001). The changing epidemiology of *Staphylococcus aureus*?. Emerg. Infect. Dis..

[8] David M.Z., Daum R.S. (2010). Community-associated methicillin-resistant *Staphylococcus aureus*: Epidemiology and clinical consequences of an emerging epidemic. Clin. Microbiol. Rev..

[9] Pitout J.D.D., Nordmann P., Laupland K.B., Poirel L. (2005). Emergence of Enterobacteriaceae producing extended-spectrum beta-lactamases (ESBLs) in the community. J. Antimicrob. Chemother..

[10] Robotham J.V., Deeny S.R., Fuller C., Hopkins S., Cookson B., Stone S. (2016). Cost-effectiveness of national mandatory screening of all admissions to English National Health Service hospitals for meticillin-resistant *Staphylococcus aureus*: A mathematical modelling study. Lancet Infect. Dis..

[11] Hubben G., Bootsma M., Luteijn M., Glynn D., Bishai D., Bonten M., Postma M. (2011). Modelling the costs and effects of selective and universal hospital admission screening for methicillin-resistant *Staphylococcus aureus*. PLoS ONE.

[12] Deeny S.R., Cooper B.S., Cookson B., Hopkins S., Robotham J. (2013). V Targeted versus universal screening and decolonization to reduce healthcare-associated meticillin-resistant *Staphylococcus aureus* Infection. J. Hosp. Infect..

[13] Jain R., Kralovic S.M., Evans M.E., Ambrose M., Simbartl L.A., Obrosky D.S., Render M.L., Freyberg R.W., Jernigan J.A., Muder R.R. (2011). Veterans Affairs initiative to prevent methicillin-resistant *Staphylococcus aureus* infections. N. Engl. J. Med..

[14] Borg M.A., Suda D., Scicluna E., Brincat A., Zarb P. (2021). Universal admission screening: A potential game-changer in hospitals with high prevalence of MRSA. J. Hosp. Infect..

[15] Munoz-Price L.S., Poirel L., Bonomo R.A., Schwaber M.J., Daikos G.L., Cormican M., Cornaglia G., Garau J., Gniadkowski M., Hayden M.K. (2013). Clinical epidemiology of the global expansion of *Klebsiella pneumoniae* carbapenemases. Lancet Infect. Dis..

[16] Centers for Disease Control and Prevention (2019). Antibiotic Resistance Threats in the United States, 2019.

[17] Sati H., Carrara E., Savoldi A., Hansen P., Garlasco J., Campagnaro E., Boccia S., Castillo-Polo J.A., Magrini E., Garcia-Vello P. (2025). The WHO Bacterial Priority Pathogens List 2024: A prioritisation study to guide research, development, and public health strategies against antimicrobial resistance. Lancet Infect. Dis..

[18] European Centre for Disease Prevention and Control (2025). Rapid Risk Assessment—Carbapenem-Resistant Enterobacterales—Third Update. https://www.ecdc.europa.eu/en/publications-data/carbapenem-resistant-enterobacterales-rapid-risk-assessment-third-update.

[19] Schwaber M.J., Lev B., Israeli A., Solter E., Smollan G., Rubinovitch B., Shalit I., Carmeli Y. (2011). Israel Carbapenem-Resistant Enterobacteriaceae Working Group. Containment of a country-wide outbreak of carbapenem-resistant *Klebsiella pneumoniae* in Israeli hospitals via a nationally implemented intervention. Clin. Infect. Dis..

[20] Temkin E., Bechor M., Lurie-Weinberger M.N., Keren-Paz A., Chen D., Lugassy C., Solter E., Schwaber M.J., Carmeli Y., CPE Working Group (2025). Population-based study of emergence and spread of Escherichia coli producing OXA-48-like carbapenemases, Israel, 2007–2023. Emerg. Infect. Dis..

[21] European Centre for Disease Prevention and Control (2021). Increase in OXA-244-Producing *Escherichia coli* in the European Union/European Economic Area and the UK Since 2013—First Update. https://www.ecdc.europa.eu/en/publications-data/rapid-risk-assessment-increase-oxa-244-producing-escherichia-coli-eu-eea.

[22] Zahar J.-R., Blot S., Nordmann P., Martischang R., Timsit J.-F., Harbarth S., Barbier F. (2019). Screening for intestinal carriage of extended-spectrum beta-lactamase-producing Enterobacteriaceae in critically ill patients: Expected benefits and evidence-based controversies. Clin. Infect. Dis..

[23] National Center for Infection Control, Israel Ministry of Health Sentinel Bacteria in General Hospitals, Annual Summary 2023. https://www.gov.il/BlobFolder/dynamiccollectorresultitem/bacterial_alert_2024/he/files_publications_units_infection-control_Bacterial_alert_2024.pdf.

[24] Bezabih Y.M., Bezabih A., Dion M., Batard E., Teka S., Obole A., Dessalegn N., Enyew A., Roujeinikova A., Alamneh E. (2022). Comparison of the global prevalence and trend of human intestinal carriage of ESBL-producing Escherichia coli between healthcare and community settings: A systematic review and meta-analysis. JAC Antimicrob. Resist..

[25] Huskins W.C., Huckabee C.M., O’Grady N.P., Murray P., Kopetskie H., Zimmer L., Walker M.E., Sinkowitz-Cochran R.L., Jernigan J.A., Samore M. (2011). Intervention to reduce transmission of resistant bacteria in intensive care. N. Engl. J. Med..

[26] Lin M.Y., Hayden M.K., Lyles R.D., Lolans K., Fogg L.F., Kallen A.J., Weber S.G., Weinstein R.A., Trick W.E. (2018). Prevention Epicenters Program, Centers for Disease Control and Prevention. Regional epidemiology of methicillin-resistant *Staphylococcus aureus* among adult intensive care unit patients following state-mandated active surveillance. Clin. Infect. Dis..

[27] Duerden B., Fry C., Johnson A.P., Wilcox M.H. (2015). The control of methicillin-resistant *Staphylococcus aureus* blood stream infections in England. Open Forum Infect. Dis..

[28] Nijssen S., Bonten M.J.M., Weinstein R.A. (2005). Are active microbiological surveillance and subsequent isolation needed to prevent the spread of methicillin-resistant *Staphylococcus aureus*?. Clin. Infect. Dis..

[29] Mainous A.G., Diaz V.A., Matheson E.M., Gregorie S.H., Hueston W.J. (2011). Trends in hospitalizations with antibiotic-resistant infections: U.S., 1997–2006. Public Health Rep..

[30] Fullybright R., Dwivedi A., Mallawaarachchi I., Sinsin B. (2016). Modeling and predicting drug resistance rate and strength. Eur. J. Clin. Microbiol. Infect. Dis..

[31] Bonten M.J., Weinstein R.A. (1996). The role of colonization in the pathogenesis of nosocomial infections. Infect. Control Hosp. Epidemiol..

[32] Huang S.S., Platt R. (2003). Risk of methicillin-resistant *Staphylococcus aureus* infection after previous infection or colonization. Clin. Infect. Dis..

[33] Temkin E., Solter E., Lugassy C., Chen D., Cohen A., Schwaber M.J., Carmeli Y., CPE Working Group (2024). The natural history of carbapenemase-producing Enterobacterales: Progression from carriage of various carbapenemases to bloodstream infection. Clin. Infect. Dis..

[34] Almagor J., Temkin E., Benenson I., Fallach N., Carmeli Y. (2018). on behalf of the DRIVE-AB Consortium. The impact of antibiotic use on transmission of resistant bacteria in hospitals: Insights from an agent-based model. PLoS ONE.

[35] Cohen B., Hyman S., Rosenberg L., Larson E. (2012). Frequency of patient contact with health care personnel and visitors: Implications for infection prevention. Jt. Comm. J. Qual. Patient Saf..

[36] Bredin D., O’Doherty D., Hannigan A., Kingston L. (2022). Hand hygiene compliance by direct observation in physicians and nurses: A systematic review and meta-analysis. J. Hosp. Infect..

[37] (2023). Eurostat Hospital Discharges and Length of Stay Statistics. https://ec.europa.eu/eurostat/statistics-explained/index.php?title=Hospital_discharges_and_length_of_stay_statistics.

[38] Morgan D.J., Liang S.Y., Smith C.L., Johnson J.K., Harris A.D., Furuno J.P., Thom K.A., Snyder G.M., Day H.R., Perencevich E.N. (2010). Frequent multidrug-resistant *Acinetobacter baumannii* contamination of gloves, gowns, and hands of healthcare workers. Infect. Control Hosp. Epidemiol..

[39] D’Agata E.M.C., Webb G., Horn M. (2005). A mathematical model quantifying the impact of antibiotic exposure and other interventions on the endemic prevalence of vancomycin-resistant Enterococci. J. Infect. Dis..

[40] Huang S.S., Datta R., Platt R. (2006). Risk of acquiring antibiotic-resistant bacteria from prior room occupants. Arch. Intern. Med..

[41] Passaretti C.L., Otter J.A., Reich N.G., Myers J., Shepard J., Ross T., Carroll K.C., Lipsett P., Perl T.M. (2013). An evaluation of environmental decontamination with hydrogen peroxide vapor for reducing the risk of patient acquisition of multidrug-resistant organisms. Clin. Infect. Dis..

[42] O’Fallon E., Gautam S., D’Agata E.M.C. (2009). Colonization with multidrug-resistant Gram-negative bacteria: Prolonged duration and frequent cocolonization. Clin. Infect. Dis..

[43] Scanvic A., Denic L., Gaillon S., Giry P., Andremont A., Lucet J.C. (2001). Duration of colonization by methicillin-resistant *Staphylococcus aureus* after hospital discharge and risk factors for prolonged carriage. Clin. Infect. Dis..

[44] Carmeli Y., Eliopoulos G.M., Samore M.H. (2002). Antecedent treatment with different antibiotic agents as a risk factor for vancomycin-resistant *Enterococcus*. Emerg. Infect. Dis..

[45] Wang J., Wang M., Huang Y., Zhu M., Wang Y., Zhuo J., Lu X. (2011). Colonization pressure adjusted by degree of environmental contamination: A better indicator for predicting methicillin-resistant *Staphylococcus aureus* acquisition. Am. J. Infect. Control.

[46] Donskey C.J., Chowdhry T.K., Hecker M.T., Hoyen C.K., Hanrahan J.A., Hujer A.M., Hutton-Thomas R.A., Whalen C.C., Bonomo R.A., Rice L.B. (2000). Effect of antibiotic therapy on the density of vancomycin-resistant Enterococci in the stool of colonized patients. N. Engl. J. Med..

[47] Cheng V.C.C., Li I.W.S., Wu A.K.L., Tang B.S.F., Ng K.H.L., To K.K.W., Tse H., Que T.L., Ho P.L., Yuen K.Y. (2008). Effect of antibiotics on the bacterial load of meticillin-resistant *Staphylococcus aureus* colonisation in anterior nares. J. Hosp. Infect..

